# Both haemagglutinin-specific antibody and T cell responses induced by a chimpanzee adenoviral vaccine confer protection against influenza H7N9 viral challenge

**DOI:** 10.1038/s41598-017-02019-1

**Published:** 2017-05-12

**Authors:** Xiang Wang, Weihui Fu, Songhua Yuan, Xi Yang, Yufeng Song, Lulu Liu, Yudan Chi, Tao Cheng, Man Xing, Yan Zhang, Chao Zhang, Yong Yang, Caihong Zhu, Xiaoyan Zhang, Sidong Xiong, Jianqing Xu, Dongming Zhou

**Affiliations:** 1 Institute of Biology and Medical Sciences, Soochow University, Suzhou, 215123 China; 2 Vaccine Research Center, Key Laboratory of Molecular Virology & Immunology, Institut Pasteur of Shanghai, Chinese Academy of Sciences, Shanghai, 200031 China; 3 Shanghai Public Health Clinical Center and Institutes of Biomedical Sciences, Key Laboratory of Medical Molecular Virology of Ministry of Education/Health at Shanghai Medical College, Fudan University, Shanghai, 200031 China

## Abstract

Since 2013, the outbreak or sporadic infection of a new reassortant H7N9 influenza virus in China has resulted in hundreds of deaths and thousands of illnesses. An H7N9 vaccine is urgently needed, as a licensed human vaccine against H7N9 influenza is currently not available. Here, we developed a recombinant adenovirus-based vaccine, AdC68-H7HA, by cloning the H7N9 haemagglutinin (HA) gene into the chimpanzee adenoviral vector AdC68. The efficacy of AdC68-H7HA was evaluated in mice as well as guinea pigs. For comparison, an H7N9 DNA vaccine based on HA was also generated and tested in mice and guinea pigs. The results demonstrated that both AdC68-H7HA and the DNA vaccine prime-adenovirus boost regimen induced potent immune responses in animals and completely protected mice from lethal H7N9 influenza viral challenge. A post-immunization serum transfer experiment showed that antibody responses could completely protect against lethal challenge, while a T cell depletion experiment indicated that HA-specific CD8^+^ T cells responses also contributed to protection. Therefore, both HA-specific humoral immunity and cellular immunity play important roles in the protection. These data suggest that the chimpanzee adenovirus expressing HA is a promising vaccine candidate for H7N9 virus or other influenza viral subtypes.

## Introduction

A novel, avian-origin H7N influenza virus emerged in East China in February 2013. Patients who were infected with the H7N9 virus suffered from respiratory tract infection, severe pneumonia and breathing difficulties, and even death^1^. By the end of April 2015, the H7N9 influenza virus had caused 630 laboratory-confirmed human infections with a mortality rate of more than 30%^2^. Previous findings showed that the new avian H7N9 virus was re-assorted from three other influenza viruses: H7N9, H7N3, and H9N2^3^. Human infections with H7 viruses had been reported rarely^4^, and pathogenic viruses were usually confined to H7N2, H7N3, and H7N7. There were no known human cases of influenza H7N9 reported prior to 2013. Thus, most humans are immunologically naïve to the novel avian H7N9 virus^1^.

Seasonal influenza vaccines in clinical use include inactivated influenza vaccines and live attenuated influenza vaccines^5^. However, their efficacies vary significantly among individuals by age and physical condition^6–8^. Moreover, live attenuated vaccines pose a risk of mutating back to the original, un-attenuated sequence^9^. These clinically used seasonal influenza vaccines provide limited protection against heterogeneous influenza viral infections, such as the H5N1 and H7N9 strains^10, 11^. Although inactivated H7N9 influenza vaccines can be produced rapidly^12^, the poor immunogenicity of the inactivated influenza vaccine has confined their use. Live attenuated H7N9 vaccines may show good immunogenicity and can confer protection against H7N9 viral infection^13^, but they may potentially re-assort with other influenza viruses owing to the segmented genome. Furthermore, preparing H7N9 viruses is a high-risk task and must be performed in a BSL3 lab. In addition, the process required to produce inactivated vaccines and live attenuated vaccines relies on the availability of specific pathogen-free (SPF) eggs, which are often in short supply due to the slaughter of live birds during the flu season. Therefore, novel H7N9 vaccines that are cheaper, more effective, and adjuvant-independent are urgently needed.

Chimpanzee adenovirus serotype 68 (AdC68) has been shown to be a good foreign gene carrier in both gene therapy and vaccine development owing to its high transduction efficiency, broad cell tropism, high gene expression, good genetic stability, and low seropositive rate in humans^14, 15^. Various vaccine candidates based on AdC68 have been developed for controlling many infectious diseases, including influenza^16–19^. The haemagglutinin (HA) protein is an influenza virus surface glycoprotein with good immunogenicity and antigenic variability, and it is responsible for viral attachment to the cell receptor and subsequent fusion with the host cell membrane^20^. HA can induce a high titre of total IgG, including neutralizing antibodies and binding antibodies against influenza virus. Immunization with recombinant HA alone is capable of protecting against influenza viral infection^21^. In addition, the HA subunit Flublok vaccine has been approved for human clinical use^22^. However, repeated immunizations and adjuvants are often needed to enhance the immunogenicity of the recombinant HA antigen. Here, we adopted the chimpanzee adenovirus AdC68 to express H7N9 HA (AdC68-H7HA) as a novel influenza vaccine. We compared the outcomes of the AdC68-H7HA vaccine with those of a DNA vaccine based on H7N9 HA and assessed the efficacy of a DNA prime-adenovirus boost regimen in both mouse and guinea pig models.

## Results

### Expression of transgene products

An E1-deleted replication-deficient chimpanzee Ad vector, AdC68-H7HA, was constructed to express the H7N9 HA gene, with AdC68-gp, a recombinant viral vector encoding the rabies virus glycoprotein, used as a control. As shown in Supplementary Fig. 1a, HA expression was detected by western blotting in HEK293 cells infected with AdC68-H7HA in a dose-dependent manner, with the majority of HA produced in HA0 and HA1 forms. HA0 is a precursor of HA that is cleaved into two subunits, HA1 and HA2, by host proteases. Fluorescence-Activated Cell Sorting (FACS) was performed to further analyse the rate of positive cells after adenoviral infection. As shown in Supplementary Fig. 1b, the rate of positive HA expression in infected cells reached ~90% when HEK293 cells were infected with 10^10^ virus particle(vp) AdC68-H7HA virus. These results indicated that the HA antigen could be highly expressed in AdC68-H7HA-infected cells.

### Antibody responses

**Figure 1:**
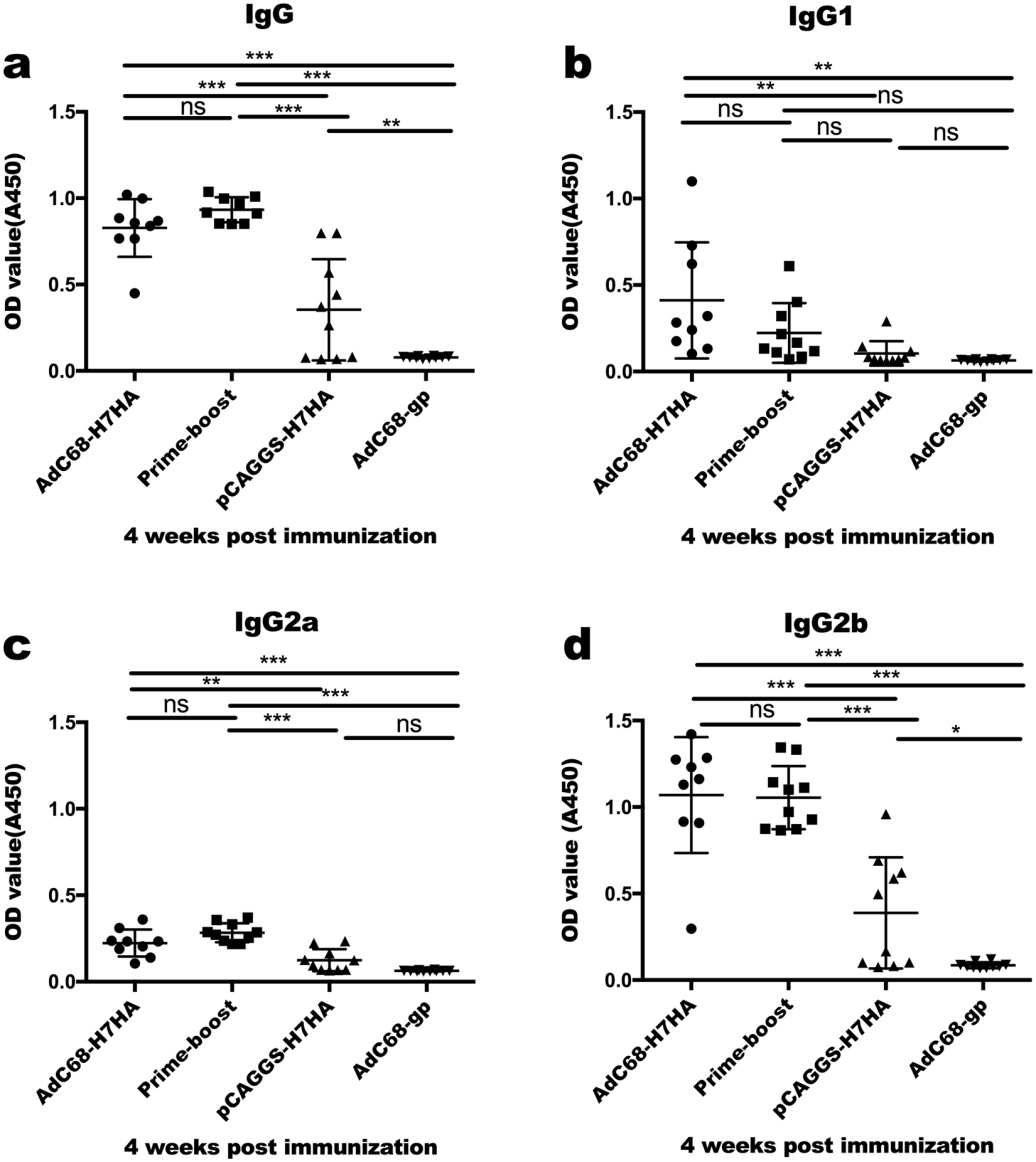
H7N9-specific IgG responses induced after immunization. C57BL/6 mice (8–10 animals/group) were divided into 4 groups: AdC68-H7HA group, pCAGGS-H7HA group, AdC68-gp group, and the DNA prime-adenovirus boost group (primed with pCAGGS-H7HA and boosted with AdC68-H7HA 2 weeks later). Four weeks after immunization, serum samples were collected for IgG detection. (**a**) IgG responses at 4 weeks after immunization. (**b**) IgG1 responses 4 weeks after immunization. **(c)** IgG2a responses 4 weeks after immunization. (**d**) IgG2b responses 4 weeks after immunization. The error bars represent the standard deviations (SD). ***p < 0.001; **p < 0.01; *p < 0.05; ns, not significant.

Four groups of mice were vaccinated with AdC68-H7HA, pCAGGS-H7HA/AdC68-H7HA (prime-boost), pCAGGS-H7HA (DNA alone), and AdC68-gp (control). ELISAs (enzyme-linked immunosorbent assays) were performed to measure HA-specific antibody responses. As shown in Fig. 1a, 4 weeks after priming, the AdC68-H7HA and prime-boost groups elicited higher IgG antibody responses than the control group, though no significant difference was observed between these two groups. The IgG responses in these two groups were maintained until 12 weeks after priming without a significant decrease (data not shown). The DNA-only group showed a weaker IgG response, though it was significantly higher than that of the control group.

To further analyse the kinetics of antibody production, IgG subtypes in sera (including IgG1, IgG2a, and IgG2b) were assessed 4 weeks after priming. As shown in Fig. 1b,c and d, approximately 50% of mice in the AdC68-H7HA group elicited high titres of IgG1; this was not statistically different from the rate in the prime-boost group but was significantly higher than rates in the DNA-only and control groups. In the prime-boost group, the level of IgG1 was low but was not significantly different from levels in the other groups. The AdC68-H7HA and prime-boost groups produced comparable titres of IgG2a and IgG2b that were significantly higher than those in the DNA-only and control groups. The DNA-only group elicited low IgG2a and IgG2b titres, with only IgG2b production being statistically higher than that in the control group. Since IgG2a and IgG2b are associated with a dominant Th1 immune response and IgG1 is indicative of a Th2 response^23^, our data indicate that AdC68-H7HA induced both Th1 and Th2 responses, whereas the prime-boost group and DNA-only group showed Th1-biased responses.

### H7N9 virus-specific CD8^+^ T cell responses

**Figure 2:**
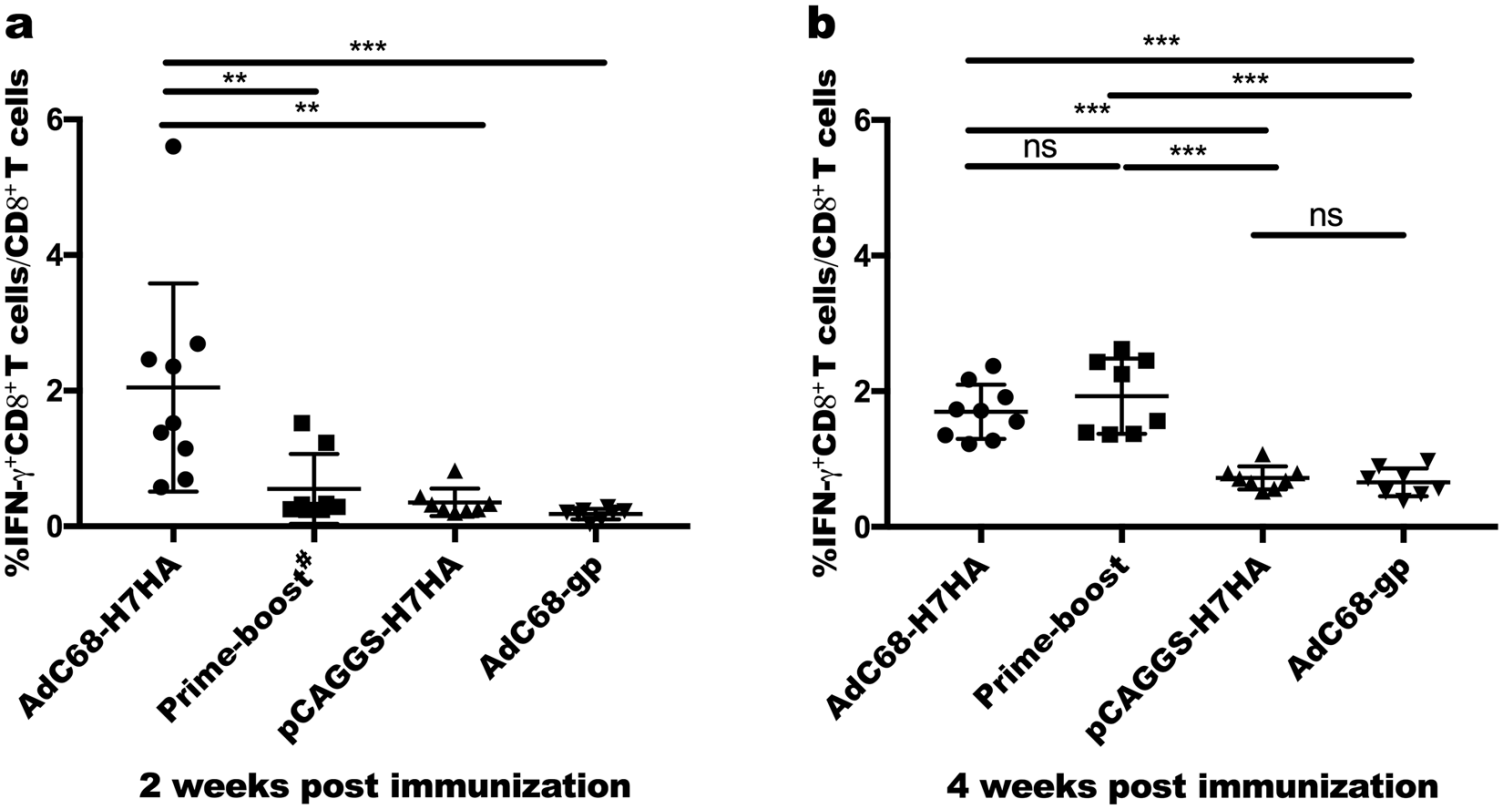
H7N9 virus-specific T cells responses. Two and 4 weeks after immunization, mouse PBMCs (peripheral blood mononuclear cells) were separated and collected. PBMCs were stimulated with H7HA peptide pools (10 μg/mL) 2 hours before adding GolgiPlug^TM^ for another 4 hours of incubation. The percentage of CD8^+^ T cells secreting IFN-γ was then measured by intracellular cytokine staining. (**a**) H7N9 virus-specific T cell responses at 2 weeks after prime immunization. (**b**) H7N9 virus-specific T cell responses at 4 weeks after prime immunization. The error bars represent the SD. ***p < 0.001; **p < 0.01; *p < 0.05. ^#^At 2 weeks post immunization, the prime-boost group received DNA priming, as in the DNA-only group.

To determine whether the tested vaccines could induce specific cellular immune responses, we examined H7N9 virus-specific CD8^+^ T cells in mice. Two or 4 weeks after prime immunization, Peripheral blood mononuclear cells(PBMCs) were harvested from the immunized mice, and functional CD8^+^ T cells were analysed by flow cytometry. Percentages of IFN-γ-secreting CD8^+^ T cells were calculated. As shown in Fig. 2a, at 2 weeks after priming, the percentage of IFN-γ-secreting CD8^+^ T cells in the AdC68-H7HA group was significantly higher than those in other groups. Four weeks after priming (Fig. 2b), the percentages of IFN-γ-secreting CD8^+^ T cells in the AdC68-H7HA and prime-boost groups were comparable, which suggests that specific T cell responses were activated after boosting in the prime-boost group and that they were significantly higher than those in the DNA-only and AdC68-gp groups. The DNA-only group induced a lower percentage of IFN-γ-secreting CD8^+^ T cells owing to its poor immunogenicity. These results indicate that the adenovirus AdC68-H7HA alone and prime-boost treatments can remarkably activate T cell responses.

### Haemagglutinin inhibition (HAI) antibody and neutralizing (NT) antibody responses

**Figure 3:**
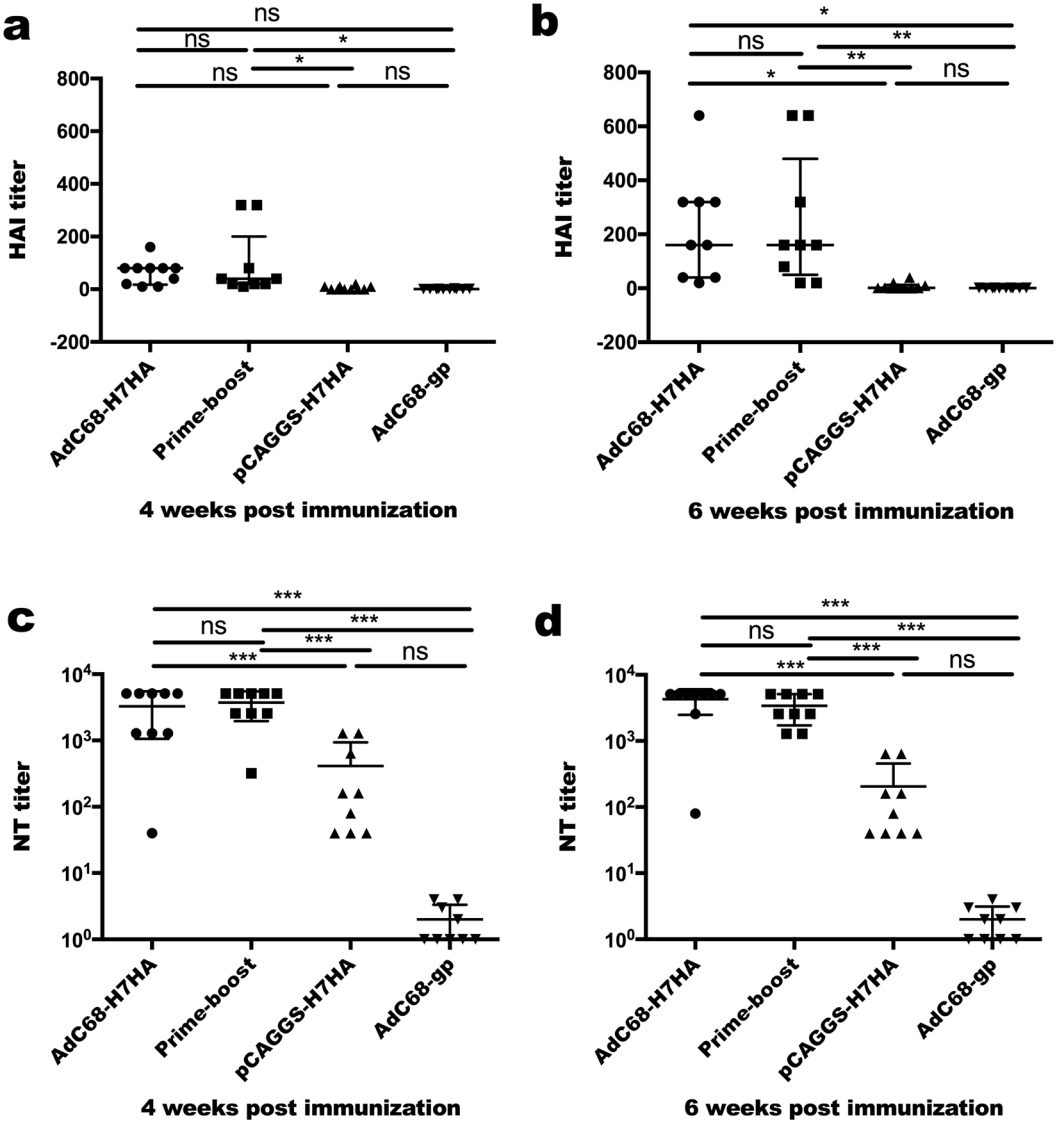
HAI and NT antibody titres of serum samples from immunized mice. C57BL/6 mice (8–10 animals/group) were divided into 4 groups: AdC68-H7HA group, pCAGGS-H7HA group, AdC68-gp group, and the prime-boost group. Four and 6 weeks after the first immunization, HAI and NT titres were detected in the serum samples. (**a,b**) HAI antibody titres against H7N9. (**c,d**) NT antibody titres against H7N9 pseudovirus. The error bars represent the SD. ***p < 0.001; **p < 0.01; *p < 0.05; ns, not significant.

HAI and NT antibodies were measured in mouse sera 4 and 6 weeks after the first immunization, as shown in Fig. 3a and b. The mean HAI antibody titre was 160 in both the AdC68-H7HA and prime-boost groups at 6 weeks after vaccination, which was higher than titres measured at week 4. However, no significant differences were observed in HAI antibody production between these two groups. In the DNA-only group, HAI titres were as low as those in the control group.

Four weeks after vaccination, the mean NT antibody titres in the AdC68-H7HA, prime-boost, and DNA-only groups were 3275, 3733, and 413, respectively. Six weeks after vaccination, the mean NT antibody titres in these groups increased to 4275, 3413, and 204 respectively, as shown in Fig. 3c and d. The NT antibody titres in the AdC68-H7HA and prime-boost groups were comparable and were significantly higher than those in the DNA-only and control groups. The NT antibody titres in the DNA-only group were significantly lower than those in the AdC68-H7HA and prime-boost groups but higher than those in the control group.

### Protection of mice from lethal challenge with the H7N9 virus

**Figure 4:**
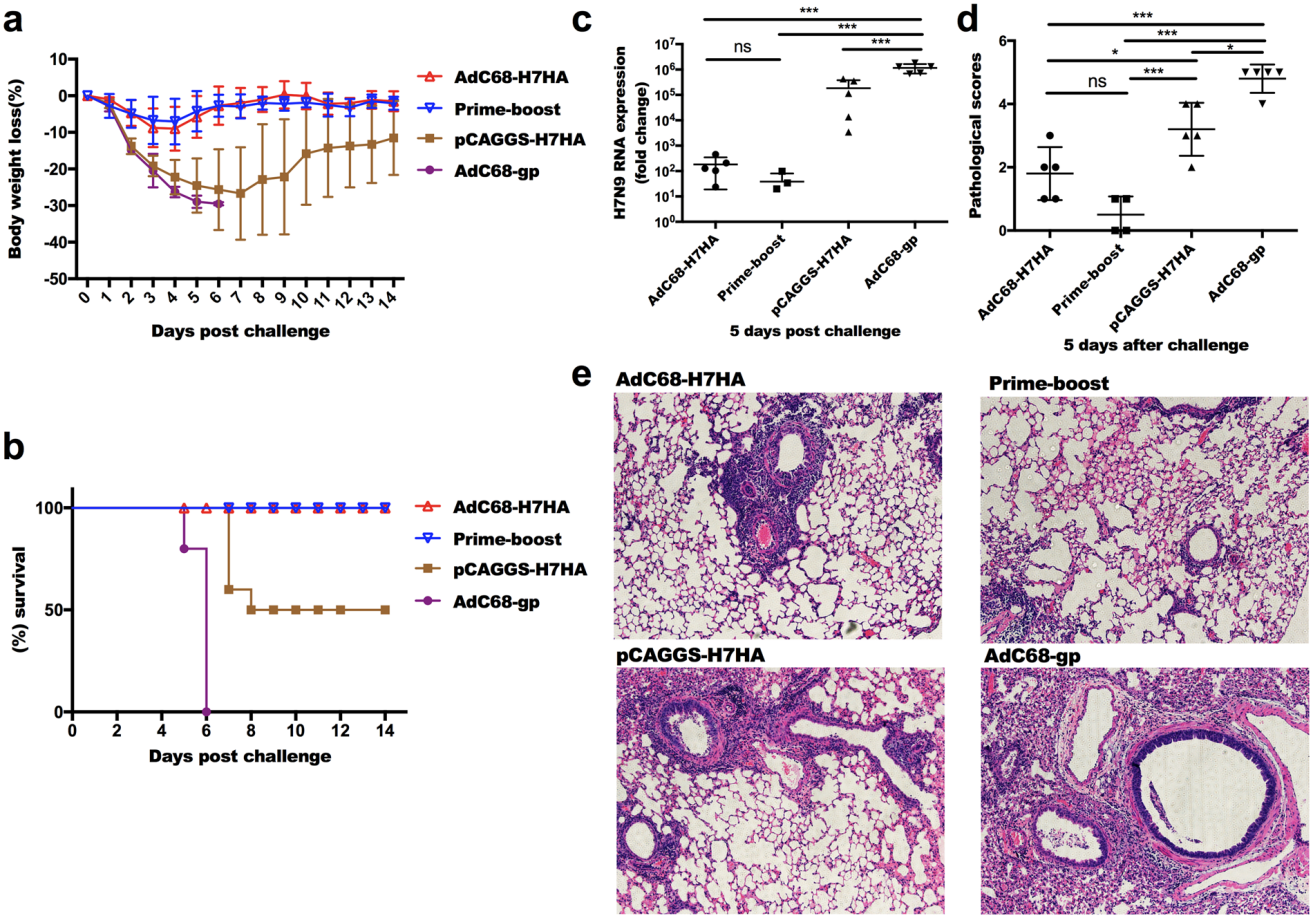
A single immunization with AdC68-H7HA or DNA prime-adenovirus boost regimen protected mice from lethal H7N9 viral challenge. Eight weeks after immunization, mice were challenged with 3.5 × 10^5^ TCID50 of H7N9 virus, after which body weights and survival rates were monitored for 14 days. (**a**) Body weight changes. (**b**) Survival rates. No significant difference was observed between the prime-boost group and the AdC68-H7HA group. Results of the prime-boost group and the AdC68-H7HA group were significantly different compared with those of the AdC68-gp group (p = 0.00001) and the DNA-only group (p = 0.03). (**c**) H7N9 viral loads in mouse lungs at 5 days post-challenge. Five days after challenge, the mice were sacrificed and the lungs were harvested and homogenized to extract the total RNA. Then, real-time PCRs were performed. (**d**) Pathological scores of the lungs at 5 days after challenge. (**e**) Lung sections were stained with H&E to assess inflammation and tissue damage on day 5 after challenge. The error bars represent the SD. ***p < 0.001; **p < 0.01; *p < 0.05; ns, not significant.

Eight weeks after immunization, mice were challenged with 3.5 × 10^5^ 50% tissue culture-infective dose (TCID50) of H7N9 virus and then monitored for 14 days. Weight loss and survival rates were analysed. As shown in Fig. 4a, the AdC68-H7HA and prime-boost groups lost less than 10% of their pre-challenge weight and then began to recover 4 days post-challenge, whereas the mean weight loss in the DNA-only group was approximately 30% of the original weight 6 days post-challenge. All mice in the control group died before or on the sixth day after challenge. The survival rates in both the AdC68-H7HA and prime-boost groups were 100%, whereas the survival rate was 50% in the DNA-only group (Fig. 4b).

### Viral loads and pathological changes in the lungs after H7N9 challenge

Five mice in each group were euthanized on the fifth day after H7N9 viral challenge. Viral loads were measured by RT-PCR analysis of lung homogenates. As shown in Fig. 4c, lung viral titres in the AdC68-H7HA group were markedly lower than those in the control group. Lung viral titres in the prime-boost group were marginally lower than those in the AdC68-H7HA group, although no statistical difference was found between these two groups. In the DNA-only group, viral titres in the mouse lung were higher than those in the AdC68-H7HA and prime-boost groups but were significantly lower than those in the control group. These results demonstrate that AdC68-H7HA, the prime-boost regimen, and the DNA vaccine all effectively reduced the viral titre in the mouse lung. Histological analysis of the lungs revealed that those in the AdC68-H7HA and prime-boost groups were close to normal, with only slight infiltration observed, and the pathology scores for both the AdC68-H7HA and prime-boost groups were lower than the DNA-only group and the control group. The DNA-only group showed severe perivascular infiltration with higher pathology scores compared with those in the AdC68-H7HA and prime-boost groups. The lungs of the control mice were seriously damaged with even more severe perivascular and interstitial infiltrates (Fig. 4d and e). Therefore, the pathology scores were consistent with the lung viral titres and pathological changes in the mouse lungs in each group.

### Immunogenicity in guinea pigs

**Figure 5:**
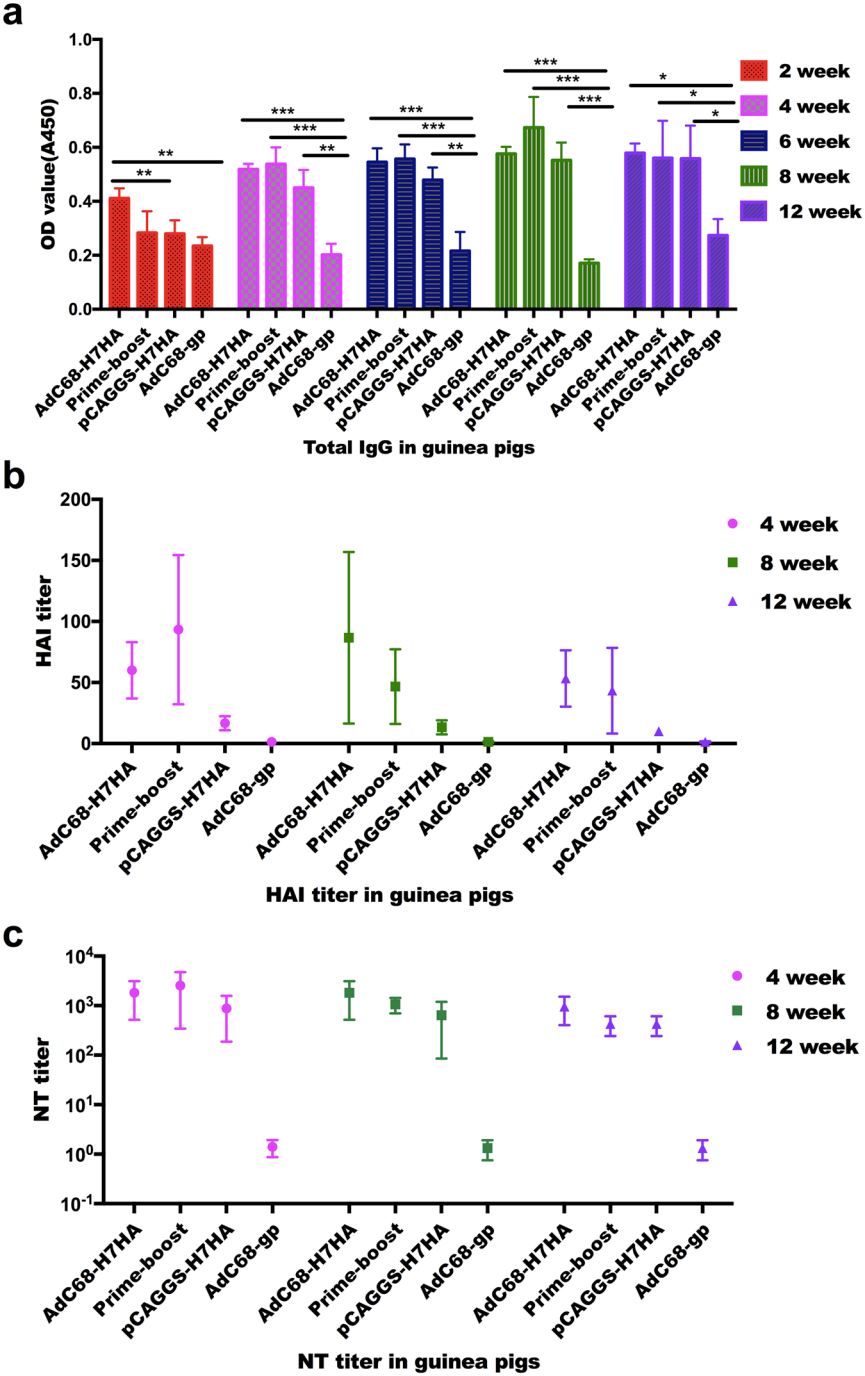
Antibody responses in guinea pigs. Twelve female guinea pigs were divided into 4 groups: the AdC68-H7HA group, the pCAGGS-H7HA group, the AdC68-gp group, and the prime-boost group. Guinea pigs were administered doses double those in mice. Serum samples were collected every 2 weeks for antibody detection. (**a**) Total IgG in guinea pigs taken at different times after immunization. (**b**) HAI titres in guinea pigs at 4, 8, and 12 weeks after immunization. (**c**) NT titres in guinea pigs at 4, 8, and 12 weeks after immunization. ***p < 0.001; **p < 0.01; *p < 0.05. ^#^At two weeks post immunization, the prime-boost group received DNA priming, as in the DNA-only group.

To evaluate the immunogenicity of the tested vaccines in guinea pigs, groups of guinea pigs were immunized intramuscularly with the same vaccination regimen as was used in the mouse study. Total serum IgG and HAI and NT antibody titres were measured. As shown in Fig. 5a, IgG was produced in all vaccine groups except for the control group at 2 weeks post-immunization and could be detected at higher levels 3 months later. The IgG titre in the AdC68-H7HA group was higher than that in the DNA-only group 2 weeks after immunization. However, no significant differences in IgG titre were found among the AdC68-H7HA, prime-boost, and DNA-only groups 4 weeks after immunization. HAI titres in the AdC68-H7HA and prime-boost groups were comparable and were significantly higher than those in the DNA-only and control groups (Fig. 5b). Similarly, NT titres in the AdC68-H7HA and prime-boost groups were comparable and were higher than those in the DNA-only and control groups (Fig. 5c). Although we found no statistically significant difference in NT titre among groups due to the small sample size, both the adenoviral vaccine and DNA vaccine elicited potent NT antibody responses in guinea pigs.

### Passive vaccination

**Figure 6:**
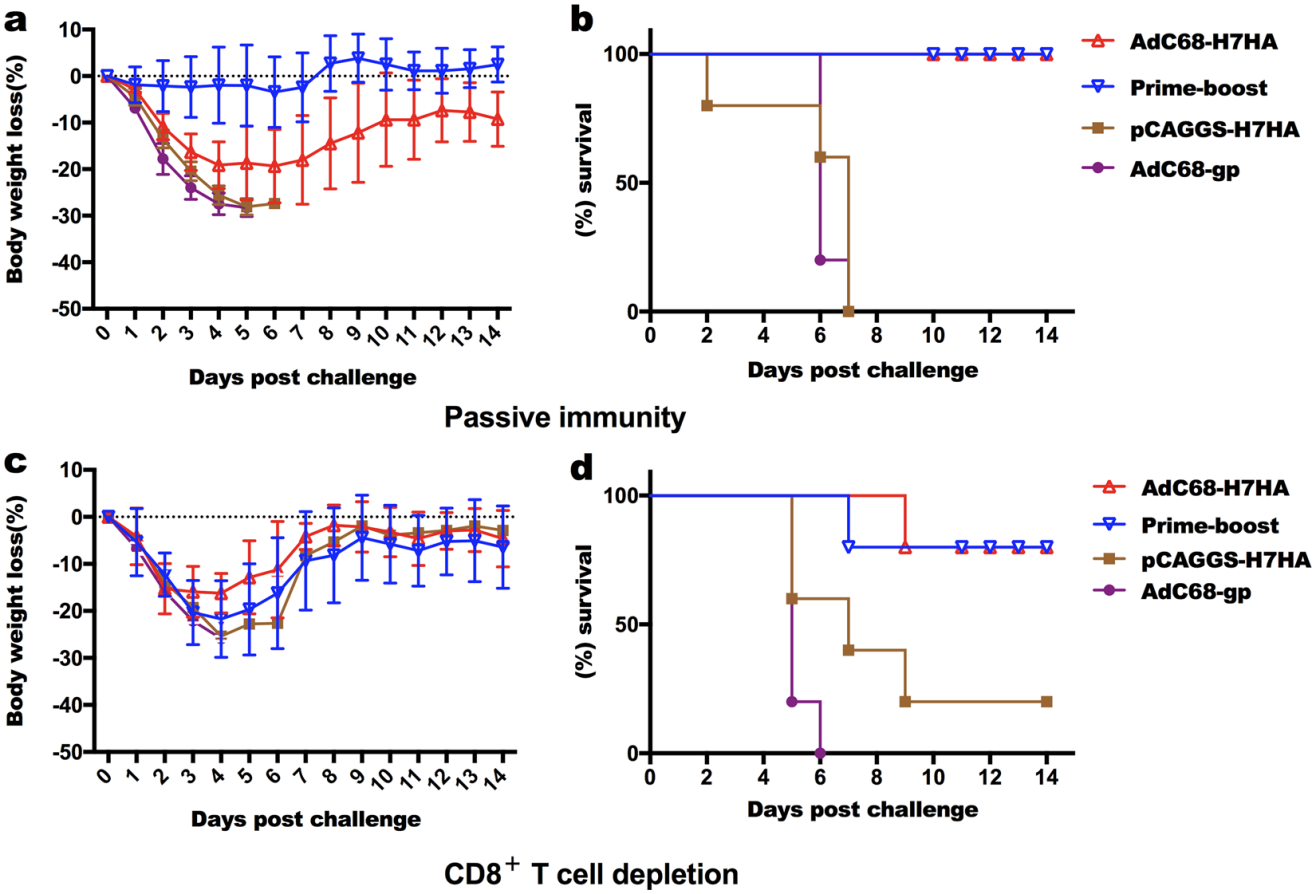
H7N9 HA-specific antibody and HA-specific CD8^+^ T cells cooperate to protect mice from lethal H7N9 viral challenge. Twenty 12-week-old female C57BL/6 mice were divided into 4 groups. The mice in each group passively received 1 mL of serum derived from immunized mice 24 hours before infection with lethal H7N9 virus. The mice in the AdC68-H7HA group and prime-boost group showed 100% protection and significant differences in body weight and survival when compared with those in the DNA-only group and the AdC68-gp group. (**a**) Body weight changes following passive immunity. (**b**) Survival rates following passive immunity. Twenty 6-week-old female C57BL/6 mice were divided into 4 groups: AdC68-H7HA group, pCAGGS-H7HA group, AdC68-gp group, and prime-boost group. At 8 weeks after immunization, mice were injected with an anti-CD8 antibody at 3 and 1 day before challenge and 1 day after challenge. Body weight changes and survival rates were monitored for 14 days. (**c**) Body weight changes after depletion of CD8^+^ T cells *in vivo*. (**d**) Survival rates after depletion of CD8^+^ T cells *in vivo*.

To determine whether the antisera induced by AdC68-H7HA or the DNA vaccine can confer protection against lethal H7N9 viral challenge, we harvested post-immunization sera from mice and passively transferred the sera to naïve mice. Twenty-four hours later, mice were challenged with the H7N9 virus. As shown in Fig. 6a, the body weights of mice in the prime-boost group remained unchanged after challenge, while the mice in the AdC68-H7HA group lost nearly 20% of their body weight and started to recover 1 week after challenge. The antisera induced by the DNA vaccine did not protect mice from H7N9 viral challenge. Taken together, these data indicate that AdC68-H7HA alone or in combination with the DNA vaccine stimulates robust antibody responses that completely protect mice from lethal H7N9 influenza challenge (Fig. 6b).

### CD8^+^ T cell depletion

To explore the role of CD8^+^ T cells in protection against H7N9 viral challenge, the immunized mice were depleted of CD8^+^ T cells *in vivo* and then challenged with H7N9 virus. As shown in Fig. 6c, depletion of CD8^+^ T cells resulted in sharp weight loss in the AdC68-H7HA and prime-boost groups, corresponding to an approximately 20% loss of the pre-challenge weight. The mice in the DNA-only group lost approximately 25% of their original weight. Both the AdC68-H7HA and prime-boost groups exhibited a survival rate of 80%, while rates in the DNA-only and control groups were 20% and 0%, respectively (Fig. 6c and d). These results demonstrate that CD8^+^ T cells also contribute to the protection of mice against H7N9 viral challenge.

## Discussion

H7N9 is a low-pathogenicity virus in birds but leads to significant morbidity and mortality in humans. Since traditional influenza vaccines cannot protect humans from H7N9 infection, the majority of the public lacks protective immunity against H7N9 viruses. In addition, the fact that the novel H7N9 virus is resistant to adamantanes and oseltamivir^24, 25^ has raised serious concerns over the ability of current antiviral agents to deal with the potential hazards. Therefore, the development of an effective vaccine has assumed the highest priority in H7N9 control and prevention. Viral vectors have proven to be very effective for gene therapy and vaccine development^26^. An H7N9 vaccine based on a modified Ankara strain vaccinia virus was effective in protecting ferrets against H7N9 lethal challenge^27^. A replication-incompetent human adenoviral vector-based influenza vaccine demonstrated that adenovirus-based expression of H7N9 HA could confer full protection against H7N9 virus challenge after two immunizations^28^. However, pre-existing neutralizing antibodies in humans have limited the clinical use of human adenoviral vectors^29^. As an alternative, chimpanzee adenoviruses have been extensively applied in vaccine development because their neutralizing antibodies are rare in humans^15, 29, 30^.

AdC68 and AdC7 are typical chimpanzee adenoviruses with immunogenicities that are similar to that of human adenovirus serotype 5^31, 32^. In this study, we developed the recombinant adenoviral AdC68-H7HA vaccine and the DNA vaccine pCAGGS-H7HA, and we compared the efficacy of AdC68-H7HA, a DNA prime-adenovirus boost regimen, and a DNA vaccine-only regimen. The results of this study demonstrated that a single immunization with AdC68-H7HA activated both humoral and cellular immunity, which provided 100% protection against H7N9 viral challenge. Total IgG was induced at an early stage and maintained at a high level for 3 months. AdC68-H7HA was sufficiently immunogenic to induce high titres of NT and HAI antibodies after a single immunization. The pathological damage to the lungs was slight, and lung viral titres were dramatically reduced compared with those in the control group. The prime-boost group also showed good immunogenicity, although no significant improvement in efficacy was observed compared with that of the single AdC68-H7HA immunization. The immunogenicity of the DNA vaccine was poor^33^; thus, it requires boosting with the same DNA vaccine or another type of vaccine. The immunogenicity of the tested vaccines was also confirmed in guinea pigs, which showed results that were consistent with those of the mouse studies.

In the present study, AdC68-H7HA and the prime-boost immunization elicited both B cell and T cell immune responses in the mouse model. Serum-transfer experiments demonstrated that the higher titres of NT antibodies elicited in these two groups conferred 100% protection against lethal challenge, suggesting that the HA-specific antibody alone could provide complete protection at a sufficiently high titre. Nevertheless, HA-specific CD8^+^ T cell responses can contribute to recovery from severe H7N9 disease and can provide broader protection against different influenza viral subtypes^34, 35^. Thus, we further validated the role of CD8^+^ T cell responses in protective immunity by conducting T cell-depletion experiments. The results indicated that without the function of CD8^+^ T cells, the degree of protection against challenge was approximately 80% in both the AdC68-H7HA and prime-boost groups, which confirmed that CD8^+^ T cells play an important role in protection.

In conclusion, in this study, we introduced and validated a recombinant H7N9 vaccine based on the chimpanzee adenoviral vector AdC68. This promises to have a profound effect on the prevention and control of the H7N9 virus. We have demonstrated that both a single immunization with the recombinant adenovirus AdC68-H7HA and the DNA prime-adenovirus boost regimen induced robust immune responses and protected mice from lethal H7N9 viral infection. Compared with the DNA vector, AdC68 is an ideal vaccine vector owing to its superior immunogenicity, and both HA-specific humoral and cellular immunity contribute to protection against H7N9 infection. In the future, AdC68-H7HA should be further developed as a potential H7N9 vaccine candidate.

## Methods

### Ethics statements

All animal studies were approved by the Institutional Animal Care and Use Committee (IACUC) of the Institut Pasteur of Shanghai. The study was carried out in strict accordance with the recommendations of the Guide for the Care and Use of Laboratory Animals.

### Plasmids, viruses, and cells

The *HA* gene (GenBank accession number: KJ633809.1) of H7N9 (A/Zhejiang/DTID-ZJU01/2013) was amplified from viral RNA, then cloned into to the p-Shuttle vector (Clontech) and subcloned into the E1-deleted chimpanzee adenoviral vector AdC68, which was generated in our lab. To rescue the recombinant adenovirus, the AdC68-H7HA vector was linearized by *Pac*I digestion and then transfected into human embryonic kidney cells (HEK293). Rescued adenoviruses were further expanded and purified by caesium chloride density-gradient centrifugation. The adenovirus concentration was measured by spectrophotometry at an absorbance of 260 nm. An AdC68 vector expressing the rabies virus glycoprotein (AdC68-gp) was constructed by the same method and used as a control virus in this study. To create the DNA vaccine, the H7N9 *HA* gene was cloned into the pCAGGS vector (Addgene). An H7N9 influenza virus (Influenza A/Shanghai/4664T/2013[H7N9]) provided by the Shanghai Public Health Clinical Center of Fudan University (China) was used as the challenge virus. HEK293 and MDCK cells were maintained in complete Dulbecco’s modified Eagle’s medium supplemented with 10% foetal bovine serum (HyClone) and 2% penicillin and streptomycin (HyClone) and cultured at 37 °C and 5% CO_2_.

### Detection of AdC68-H7HA expression by western blotting and FACS

HA expression was detected by western blotting and FACS analysis. HEK293 cells were infected with different quantities of adenovirus (10^8^, 10^9^, or 10^10^ vp) and 24 hours later, cells were harvested and lysed with RIPA Lysis Buffer (Beyotime). Cell lysates were analysed by western blotting with an anti-H7N9 polyclonal antibody (Sino Biological). For the FACS experiments, HEK293 cells were harvested at 24 hours post-infection, washed in phosphate-buffered saline (PBS), incubated with the H7N9 polyclonal antibody for 2 hours, washed twice in PBS, and stained with a fluorescein isothiocyanate (FITC)-conjugated anti-mouse IgG. FACS was performed with a Fortessa flow cytometer (Becton Dickinson), and the data were analysed with FlowJo 10.0.6 software (Tree Star).

### Animal studies

Six- to eight-week-old female C57BL/6 mice were purchased from the Shanghai Laboratory Animal Center (China) and housed at the Institut Pasteur of Shanghai Animal Facility. The mice were divided into four groups, which were immunized intramuscularly with AdC68-H7HA (5 × 10^10^ vp), pCAGGS-H7HA (50 µg), pCAGGS-H7HA (50 µg), or AdC68-gp (5 × 10^10^ vp). Two weeks after the prime, the second group only was boosted with AdC68-H7HA at a dose of 5 × 10^10^ vp, which was designated as the prime-boost group. All mice were bled every 2 weeks. Two months after vaccination, the mice were challenged with 3.5 × 10^5^ TCID50 of the H7N9 virus. Five mice in each group were euthanized on the fifth day post-challenge to check the viral titres and assess pathological changes in the lungs. For the remaining 10 mice in each group, the body weights and survival rates were monitored daily for 14 days post-challenge, and the mice were euthanized when they lost over 30% of their pre-challenge body weight. All experiments related to the H7N9 virus were conducted in a biosafety level 3 laboratory following the standard operating protocols approved by the Institutional Biosafety Committee at the Shanghai Public Health Clinical Center, Fudan University.

To evaluate vaccine immunogenicity in guinea pigs, 12 female guinea pigs weighing 250–280 g each were purchased from the Shanghai Laboratory Animal Center (China) and housed at the Institut Pasteur of Shanghai Animal Facility, which was approved by the IACUC. The guinea pigs were divided into four groups and received the same vaccination regimen as the mice but were administered twofold higher dosages. Blood samples were collected every 2 weeks to harvest sera until 3 months after priming. The guinea pigs were euthanized after the last serum collection.

### Antibody measurements and isotyping

To measure specific antibody responses and IgG subtypes in the serum samples, ELISAs were performed as previously described^36^. Briefly, 96-well flat ELISA plates (Costar) were coated with 50 μL of inactivated, purified H7N9/AH/1/13-PR8 virus (50 ng/well) and incubated for 3 hours at 37 °C. The plates were blocked with 100 μL 5% skim milk at 37 °C for 2 hours. The plates were then washed 3 times with PBST (PBS with 0.5% Tween 20). Antisera were diluted 1:100 with PBS and added to the plates. Plates were washed, a 1:10,000 dilution of horseradish peroxidase (HRP)-conjugated goat anti-mouse IgG (Sigma-Aldrich) was then added to each plate, and the plates were incubated for 1 hour at 37 °C. After the final wash, 50 μL 3,3′,5,5′-tetramethylbenzidine (TMB) (New Cell & Molecular Biotech) was added to each plate for 5 min. The reaction was stopped by adding 50 μL 2 M H_3_PO_4_ to each well. The plates were read at 450 nm using a Varioskan Flash multimode reader (Thermo Scientific). All samples were tested in triplicate. For the isotyping experiments, we used HRP-conjugated secondary antibodies against mouse IgG1, IgG2a, and IgG2b (Southern Biotechnology).

### Intracellular staining of cytokines

Four groups of mice were immunized as described above. Mouse PBMCs were collected two weeks after prime and boost immunizations. To detect intracellular cytokine production, 2 × 10^6^ PBMCs were stimulated with an H7N9 HA peptide pool (10 μg/ml) for 2 hours. GolgiPlug^TM^ was then added, and the cells were incubated for another 4 hours. Then, cells were surface-stained with anti-TCR β chain, anti-CD4, and anti-CD8 antibodies (BD Bioscience), followed by intracellular staining with anti-IFN-γ (BD Bioscience). Samples were analysed using a Fortessa Flow cytometer (BD Bioscience), and the data were analysed using FlowJo 10.0.6 software (Tree Star).

### Haemagglutination-inhibition (HAI) assay

All serum samples were treated with a receptor-destroying enzyme from *Vibrio cholerae* (Denka Seiken) at 37 °C overnight and later heat-inactivated at 56 °C for 30 min. The presence of HAI antibody was determined using four haemagglutination units from each inactivated H7N9 virus and 0.5% red blood cells (RBCs). The HAI titre was defined as the reciprocal of the highest dilution of serum that completely prevented RBC agglutination.

### Micro-neutralisation assay

A pseudovirus with the HA and neuraminidase (NA) membrane proteins from influenza A/Shanghai/4664T/2013(H7N9) and the capsid protein from HIV was generated as previously described^37^, and then a micro-neutralization assay was performed as follows. In a 96-well plate, twofold serially diluted serum samples beginning at 1:40 were incubated with 200 median TCID50 of pseudovirus in a final volume of 100 μL at 37 °C for 1 hour. Then, the mixture was added to cultured MDCK cells. After incubation overnight, the cells were washed with 200 μL of PBS and cultured in complete Dulbecco’s modified Eagle’s medium for 48 hours in the original 96-well plate. The relative luciferase activity (RLA) was measured using the BrightGlo luciferase substrate (Promega). The percent inhibition was calculated as follows: (RLA in the virus-challenge control – RLA in the test well for each serum at specific dilution)/RLA in the virus-challenge control. The 50% inhibitory concentration (IC_50_) titre was determined as the reciprocal of the highest dilution that resulted in >50% reduction in luciferase activity.

### Viral loads in lungs

Mice were euthanized 5 days after challenge. Lung tissues were used for total RNA extraction. Viral loads in lungs were analysed by quantitative real-time reverse transcription-PCR (RT-PCR), using the One-Step RT-PCR Kit (TaKaRa). The thermocycling conditions used were as follows: 42 °C for 10 min followed by 95 °C for 1 min and 45 cycles of 95 °C for 15 s and 60 °C for 45 s. All data were analysed using the REALPLEX2.2 software (Eppendorf). The housekeeping gene glyceraldehyde phosphate dehydrogenase (*GAPDH*) was used as an internal reference gene. The results are represented as relative expression levels compared to those in the control group. Primers and probes (5′-FAM; 3′-BHQ1) with the following sequences were used for determining influenza viral loads: H7N9: F-5′-GAAGAGGCAATGCAAAATAGAATACA-3′, R-5′-CCCGAAGCTAAACCARAGTATCA-3′, Probe-5′-CCAGTCAAACTAAGCAGYGGCTACAAA-3′; GAPDH: F-5′-CAATGTGTCCGTCGTGGATCT-3′, R-5′-GTCCTCAGTGTAGCCCAAGATG-3′, Probe-5′-CGTGCCGCCTGGAGAAACCTGCC-3′.

### Histology

Mouse lung tissues were dissected 5 days post-challenge and fixed in 4% formaldehyde for 24 hours at 4 °C, after which haematoxylin and eosin (H&E) staining was performed as previously described^36^. Histopathological changes were scored with the following criteria: a score of 1 indicated no pathology, 2 indicated perivascular infiltrates, 3 indicated perivascular and interstitial infiltrates affecting <20% of the lobe section, 4 indicated perivascular and interstitial infiltrates affecting 20 to 50% of the lobe section, and 5 indicated perivascular and interstitial infiltrates affecting >50% of the lobe section.

### Passive vaccination

Four groups of C57BL/6 mice were immunized with the same vaccination regimen used in the mouse studies described above to make high-titre immune sera. Serum samples were collected from mice 8 weeks after priming. Twenty 12-week-old female C57BL/6 mice were divided into 4 groups of recipient mice, which were intravascularly injected with 1 mL of corresponding antisera. Twenty-four hours later, mice were challenged with 3.5 × 10^5^ TCID50 of H7N9 virus. Body weights and survival rates were monitored daily post-challenge for 14 days, and the mice were euthanized when they lost >30% of their pre-challenge body weight.

### *In vivo* depletion of CD8^+^ T cells

Four groups of C57BL/6 mice were immunized with the same vaccination regimen used in the mouse studies described above. Eight weeks after priming, CD8^+^ T cells in all mice were depleted by intravenous (i.v.) injection with 0.4 mg of a rat anti-mouse CD8a monoclonal antibody (2.43, IgG2b; BioXcell) at 3 days before, 1 day before, and 1 day after challenge^38^. The efficiency of CD8^+^ T depletion measured on day 3 post-challenge was 99% based on FACS analysis of lymphocytes in blood. All mice were infected with 3.5 × 10^5^ TCID50 of H7N9 virus. Body weights and survival rates were monitored daily post-challenge for 14 days, and mice were euthanized when they lost >30% of their pre-challenge body weight.

### Statistical analysis

Antibody titres, T cell responses, and viral titres were compared among groups with one-way ANOVA. Fisher’s exact test was performed to compare the number of deaths among groups. P-values < 0.05 were considered statistically significant. GraphPad Prism software v6.0 (GraphPad) was used for statistical analysis.

## Electronic supplementary material

Expression of the target protein H7N9 HA in HEK293 cells
