# Supplementary material for: Both haemagglutinin-specific antibody and T cell responses induced by a chimpanzee adenoviral vaccine confer protection against influenza H7N9 viral challenge
Source: Sci Rep. 2017 May 12;7:1854. doi: 10.1038/s41598-017-02019-1 (PMC5431854; doi:10.1038/s41598-017-02019-1)
Supplement: Supplementary file 1 — Expression of the target protein H7N9 HA in HEK293 cells [file 41598_2017_2019_MOESM1_ESM.pdf]

Supplementary information：

# Both haemagglutinin-specific antibody and T cell responses induced by a chimpanzee adenoviral vaccine confer protection against influenza H7N9 viral challenge

Authors: Xiang Wang[1,2,†], Weihui Fu[3,†], Songhua Yuan[3], Xi Yang[2], Yufeng Song[2], Lulu Liu[1,2], Yudan Chi[2], Tao Cheng[2], Man Xing[2], Yan Zhang[2], Chao Zhang[2], Yong Yang[2], Caihong Zhu[2], Xiaoyan Zhang[3], Sidong Xiong[1,*] Jianqing Xu[3,*], and Dongming Zhou[2,*]

Affiliations:

[1]Institute of Biology and Medical Sciences, Soochow University, Suzhou 215123, China

[2]Vaccine Research Center, Key Laboratory of Molecular Virology & Immunology, Institut Pasteur of Shanghai, Chinese Academy of Sciences, Shanghai 200031, China

[3]Shanghai Public Health Clinical Center and Institutes of Biomedical Sciences, Key Laboratory of Medical Molecular Virology of Ministry of Education/Health at Shanghai Medical College, Fudan University, Shanghai 200031, China

*Correspondence and requests for materials should be addressed to: Dongming Zhou, dmzhou@sibs.ac.cn; Jianqing Xu, xujianqing2014@126.com; Sidong Xiong, sdxiong@suda.edu.cn

† Equal contributors

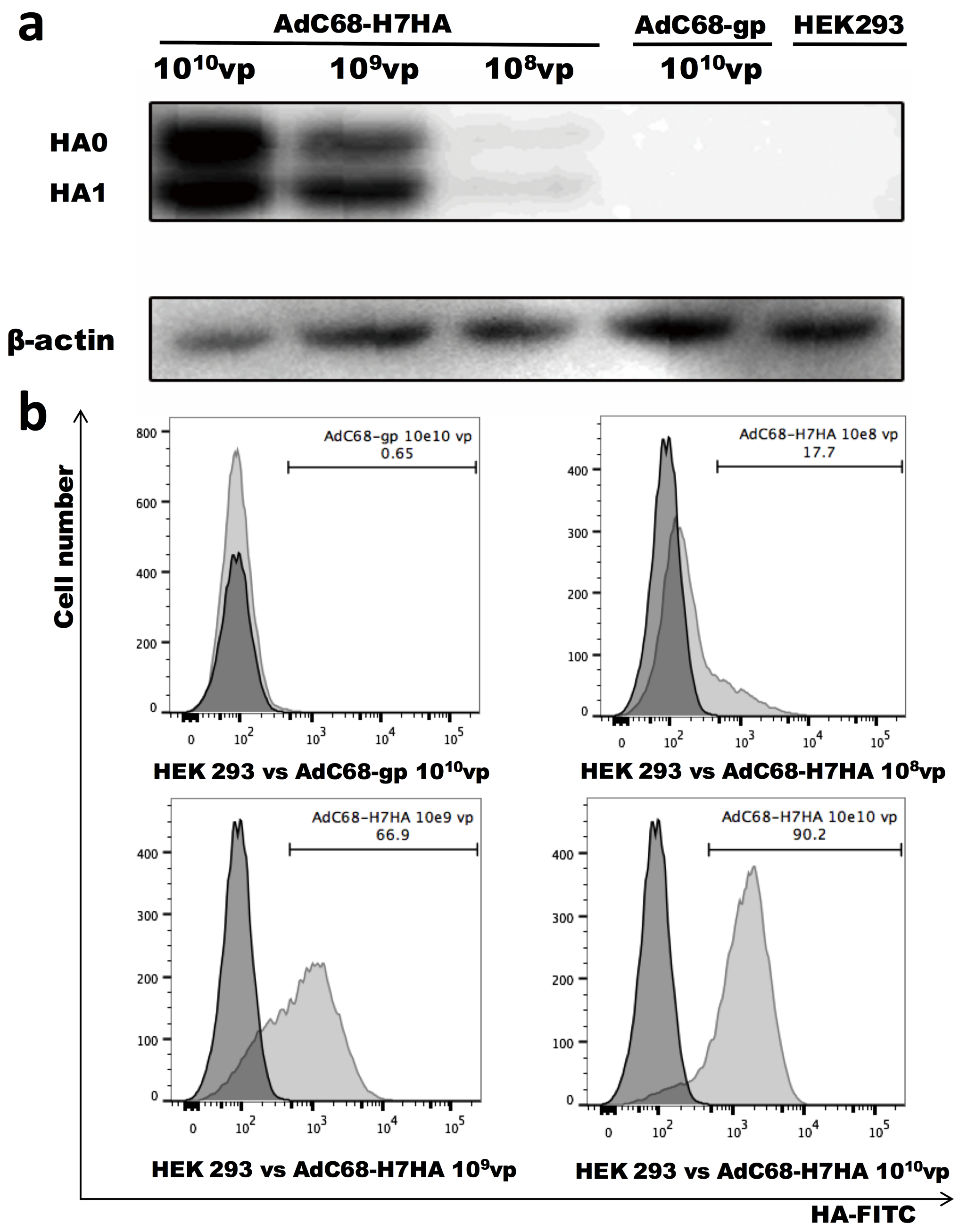

Supplementary Figure 1. Expression of the target protein H7N9 HA in HEK293 cells. **(a)** Different quantities of AdC68-H7HA (10^{10}, 10^9, or 10^8 vp) were used to infect HEK293 cells in a 6-well plate. AdC68-gp (10^9 vp) was used a negative control, and uninfected HEK293 cells were used as blank control. Twenty-four hours later, cell lysates were analyzed by western blotting using an anti-H7 HA polyclonal antibody. **(b)** At 24 hours after infection with AdC68-H7HA or AdC68-gp, the HEK293 cells were resuspended, washed, incubated with mouse H7 HA-positive sera, and then stained with a FITC-conjugated anti-mouse antibody. The percentage of positive HEK293 cells in each group was determined by FACS analysis.
